# The impact of a growth mindset on high school students’ learning subjective well-being: the serial mediation role of achievement motivation and grit

**DOI:** 10.3389/fpsyg.2024.1399343

**Published:** 2024-07-17

**Authors:** Wei Zhao, Xiang Shi, Mingfei Jin, Yangyang Li, Chen Liang, Yilong Ji, Jiaxuan Cao, Mohamed Oubibi, Xiaolin Li, Yuan Tian

**Affiliations:** ^1^School of Education, Zhejiang Normal University, Jinhua, China; ^2^School of Educational Sciences, Liaocheng University, Liaocheng, China; ^3^School of Psychology, Zhejiang Normal University, Jinhua, China; ^4^Smart Learning Institute, Beijing Normal University, Beijing, China; ^5^College of Education, Philippine Women’s University, Manila, Philippines; ^6^Liaocheng Second Experimental Primary School, Liaocheng, China

**Keywords:** growth mindset, learning subjective well-being, achievement motivation, grit, high school students

## Abstract

**Purpose:**

The learning subjective well-being of high school students has significant value for their academic achievement and future life development. A growth mindset is one of the key factors affecting the learning subjective well-being of high school students. However, research on the mechanism by which a growth mindset affects learning subjective well-being is still relatively limited. Therefore, the study aims to investigate the impact of a growth mindset on the learning subjective well-being of high school students, as well as the role that achievement motivation and grit play as serial mediators in this relationship.

**Methods:**

This study employed a convenience sampling method to select 708 high school students from Chinese public high schools as participants. The research utilized the Growth Mindset Scale, Achievement Motivation Scale, Grit Scale, and the Learning Subjective Well-being Questionnaire for High School Students to collect data. All data were analyzed using SPSS 26.0, employing Model 6 from Hayes’ SPSS PROCESS macro to test the serial mediation model.

**Results:**

Our results found that (1) high school students’ growth mindset positively predicted their learning subjective well-being. (2) Achievement motivation played a mediating role between a growth mindset and learning subjective well-being among high school students. (3) Grit acted as a mediator between learning subjective well-being and growth mindset among high school students. (4) Achievement motivation and grit served as serial mediators between a growth mindset and learning subjective well-being among high school students.

**Conclusion:**

A growth mindset can influence the learning subjective well-being of high school students through achievement motivation and grit. Educators can enhance the learning subjective well-being of high school students by implementing intervention strategies that foster a growth mindset, achievement motivation, and grit.

## 1 Introduction

High school is considered the golden age for students to learn and develop (Eryılmaz, 2012; Yu et al., 2023). Students’ attitudes toward learning during this time are greatly influenced by their subjective well-being (Konu et al., 2002). A strong sense of learning subjective well-being can help students develop a passion for learning, boost their self-assurance in academic pursuits, and lay the groundwork for both academic success and a happy future (Long et al., 2012; Liu et al., 2015). However, many Chinese students currently feel bored and burdened with increasingly heavy schoolwork and pressure during their learning process (Kirkpatrick and Zang, 2011; Liu and Lu, 2011, 2012). This negative cycle leads many students to become perfunctory in their studies and deal with situations of exhaustion (Jiang et al., 2021; Lu et al., 2021; Fu et al., 2022). Therefore, it is particularly important to improve the learning subjective well-being of high school students and thoroughly investigate the factors that influence it (Bücker et al., 2018). Mindset has consistently been a prominent research topic in the fields of psychology and education (Kim and Park, 2021). Recently, particular attention has been given to the growth mindset as it is thought to have a big impact on students’ learning subjective well-being (Macnamara and Burgoyne, 2023). However, there is a dearth of data regarding the effects of a growth mindset on learning subjective well-being of high school students. The “other” aspects, such as family and school environments (Goswami, 2012; Lampropoulou, 2018), parents and their parenting styles (Li R. et al., 2020), learning and academic performance (Liu and Zhang, 2006), as well as the personality and emotional experiences (Ortiz Alvarado et al., 2019), are the main focus of current research on the learning subjective well-being of high school students, with less attention paid to the growth mindset of high school students (King and Trinidad, 2021). Furthermore, drawing from existing research evidence and theories, achievement motivation and grit may serve as potential mediating mechanisms between a growth mindset and learning subjective well-being (Liu et al., 2021; Zhao et al., 2022). Therefore, this study aims to explore in detail the impact of a growth mindset on the learning subjective well-being of high school students, while examining the mediating role of achievement motivation and grit. This study not only closes the gap in the existing literature but also offers recommendations for raising high school students’ subjective well-being in the learning process.

### 1.1 The concept of a growth mindset and current research status

A growth mindset is proposed by Dweck (2006). Initially, she studied the innate concepts of intelligence, which later changed to the theory of mindset (Wu, 2021). This theory suggests two mindsets, among which a growth mindset is one of the basic beliefs that individuals hold about their intelligence or abilities, believing that intelligence or abilities can continuously develop and change as a result of evolving experiences and education. In contrast, a fixed mindset posits that intelligence or ability is static and difficult to change (Dweck, 2006; Zhao et al., 2018). According to the Social Intuitionist Theory, people use their beliefs to predict and select their own behavior by judging and explaining the objects and phenomena in their environment (Heider, 1958). People who have a growth mindset frequently think that ability develops incrementally, believing that abilities are variable and can be developed (Dweck, 2006; Diao et al., 2020; Zhao et al., 2022). They will proactively learn to reach their full potential, exhibit bravery in the face of academic difficulties, persevere in the face of setbacks, and choose to put in more effort to achieve their goals (Zhao et al., 2022).

The research on a growth mindset in academia mainly focuses on two aspects. First, in the field of education, there is considerable attention on the impact of a growth mindset on students’ academic performance, instructional design, and teaching reforms (Wang, 2017; Yeager et al., 2019; Niu and Li, 2022). These studies explore strategies for cultivating a growth mindset and promote related intervention programs to unlock students’ potential (Paunesku et al., 2015; Li and Geng, 2018). Second, researchers have investigated factors influencing students’ growth mindset and academic adaptation. For example, Ma et al. (2019) found a significant positive correlation between parental autonomy support and a growth mindset, while Zhao et al. (2022) revealed significant positive correlations between a growth mindset, future time perspective, and grit

### 1.2 The relationship between a growth mindset and learning subjective well-being

The overall evaluation of a person’s quality of life based on their own standards is known as subjective well-being (Diener, 1984; Ding et al., 2021). In the context of learning, learning subjective well-being refers to how students feel about their overall learning experience, which typically includes the pleasure, fulfillment, and sense of accomplishment they feel before, during, or after learning (Gan and Lai, 2010; Song et al., 2010).

According to the mindset theory (Dweck, 2006), individuals with a growth mindset tend to believe that abilities can be continuously developed. Consequently, they invest more effort in learning to enhance their abilities. They focus less on outcomes and more on improving their skills during the learning process. They possess a strong sense of self-efficacy toward learning, enabling them to respond positively even in the face of failure, thereby achieving a positive learning experience (Zeng et al., 2019; Liu et al., 2021). However, there is currently relatively little research on the relationship between a growth mindset and learning subjective well-being among high school students. Research has found that individuals with higher levels of a growth mindset tend to have stronger subjective well-being (Ortiz Alvarado et al., 2019; Liu et al., 2021). Therefore, it can be speculated that learning subjective well-being, a specific manifestation of subjective well-being in the context of learning (Wang and Han, 2018), may also be influenced by a growth mindset. As such, this study put forth hypothesis 1: A growth mindset would positively predict the learning subjective well-being of high school students.

### 1.3 The mediating role of achievement motivation between a growth mindset and learning subjective well-being

Researchers have investigated how a growth mindset affects learning subjective well-being and achievement motivation (Liu et al., 2021; Zhao et al., 2022). Achievement motivation is the inner drive that propels people to strive for greatness and success, including the desire to succeed and stay away from failure (Yang et al., 2016). According to the intelligence achievement motivation model proposed by Dweck (2006), individuals with different mindsets set different achievement goals. Students with a growth mindset establish mastery goals, while those with a fixed mindset set performance goals. These different goal orientations generate distinct motivations. Research has found that a person’s growth mindset significantly influences their drive for achievement; those who have a higher growth mindset are also more driven to succeed (Liu and Cai, 2011).

The goal theory of happiness holds that achieving goals or meeting personal needs results in the development of subjective well-being (Brunstein and Schmitt, 2004). Achievement motivation is closely related to a person’s needs for self-realization and success, and it may also influence learning subjective well-being. According to certain studies, people with a stronger motivation to pursue success have higher subjective well-being; conversely, those with a stronger motivation to avoid failure have lower subjective well-being (Elliot et al., 1997, 2006; Song et al., 2015; Gao and Bai, 2021). Based on this, this study proposed hypothesis 2: The relationship between a growth mindset and learning subjective well-being of high school students would be mediated by achievement motivation.

### 1.4 The mediating role of grit between a growth mindset and learning subjective well-being

Grit may also play a mediating role between a growth mindset and learning subjective well-being. According to Duckworth et al. (2007), grit is a psychological trait characterized by a person’s persistence in pursuing long-term goals over an extended period of time and their continued demonstration of a strong interest in or enthusiasm for these goals. As a trait of individual differences, grit is typically classified into two categories: persistence and consistency of interest (Wei and Hu, 2017). People who have a growth mindset usually do not give up easily when facing setbacks and failures. They persist in striving, face setbacks head-on, actively seek strategies to solve problems, and derive a sense of pleasure from the process (Song and Xu, 2019). This is exactly how grit manifests in individuals, and studies have shown a strong relationship between grit across various domains and a growth mindset (Zhao et al., 2018, 2022; Park et al., 2020).

Meanwhile, the character strengths theory posits that positive personality traits (such as grit) exert a beneficial impact by influencing individuals’ cognition, emotions, and behaviors (Wagner and Ruch, 2015; Jiang et al., 2018). People with grit personalities are better able to handle obstacles and failures in life, generating more positive emotional experiences (Liu, 2020). Research has confirmed a significant correlation between grit and subjective well-being, with grit positively predicting subjective well-being (Liu et al., 2021). These findings imply that the relationship between learning subjective well-being and a growth mindset may be significantly mediated by grit. Thus, this study proposed hypothesis 3: Grit would act as a mediator between a growth mindset and the learning subjective well-being of high school students.

### 1.5 The serial mediation role of achievement motivation and grit

Achievement motivation and grit, as hot topics in the field of education research, are receiving increasing attention (Nota et al., 2004; Li X. et al., 2020; Wang et al., 2020). This study aims to investigate how these two factors mediate the relationship between a growth mindset and learning subjective well-being. According to the perspective of achievement motivation theory (McClelland, 1961), individuals with a strong need for achievement aspire to accomplish tasks with greater perfection and attain greater success. They seek the enjoyment of overcoming difficulties and striving for success, as well as the sense of accomplishment that follows. As an innate motivator, achievement motivation is primarily demonstrated by an individual’s perseverance and hard work in finishing significant tasks by themselves (Zhao et al., 2022). Achievement-oriented individuals tend to exhibit greater grit and are more likely to persevere in achieving long-term objectives (Wang et al., 2020). Previous studies have also confirmed that achievement motivation has a positive impact on grit. For instance, the survey by Li et al. (2021) revealed a strong positive correlation between achievement motivation and grit, and psychological independence and achievement motivation are key elements influencing the development of grit in teenagers. This indicates that achievement motivation and grit may act as serial mediators in the process of the influence of a growth mindset on the learning subjective well-being of high school students. Based on this, this study proposed hypothesis 4: Achievement motivation and grit would play a serial mediation role between a growth mindset and the learning subjective well-being of high school students.

### 1.6 The current study

**Figure 1:**
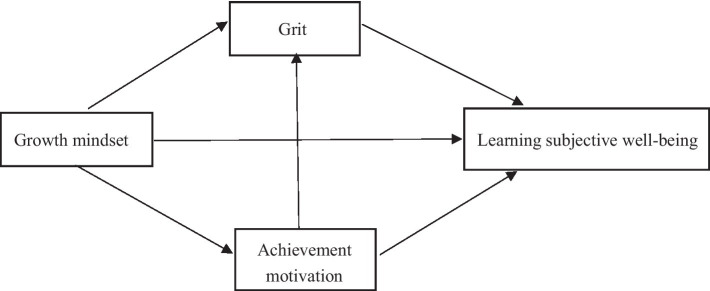
Research hypothesis model.

In summary, a growth mindset fosters a positive psychological framework that cultivates students’ achievement motivation and enthusiasm for learning, diminishes fear of failure, nurtures grit, and enhances self-efficacy, which collectively elevate the subjective well-being of high school students. This study identified the mechanisms of achievement motivation and grit in the serial mediation model (see Figure 1) that explains the influence of a growth mindset on the learning subjective well-being of high school students. This model seeks to offer a new theoretical perspective for comprehending the factors affecting the learning subjective well-being of high school students and propose feasible ways to improve the learning subjective well-being of high school students based on evidence.

## 2 Methods

### 2.1 Participants and procedures

A questionnaire was conducted among high school students in grades one, two, and three in a certain area of Shandong Province, using a convenience sampling method and class as the sampling unit. The age range of the participants was between 14 and 17 years old (15.84 ± 0.97). According to the instructions of the investigators, participants completed a questionnaire within one course in their respective classrooms, which took approximately 40 min. A total of 723 pieces of data were collected, of which 15 were deemed invalid due to careless responses, blank answers, and response times of less than 1 minute, and were therefore excluded. Ultimately, 708 valid sample data were obtained, yielding a 97.9% effectiveness rate for the questionnaire. The Ethics Committee of Zhejiang Normal University’s School of Education approved this study, which complies with the guidelines of the Helsinki Declaration, and informed consent was obtained from all participants. Table 1 displays the subjects’ basic statistical results.

**Table 1:** Basic statistics of samples.

	Frequency	Percentage
Gender		
Male	245	34.6
Female	463	65.4
Grade		
Grade 1	263	37.1
Grade 2	214	30.2
Grade 3	231	32.6
Family background		
Countryside	460	65.0
City	248	35.0
Score performance		
Excellent	174	24.6
Good	233	32.9
Average	205	29.0
Poor	96	13.6
School type		
Key high school	447	63.1
Non-key high school	261	36.9
Total	708	100.0

The distinction between key and non-key high schools lies in their classification as priority development establishments. In the context of Chinese education, this distinction is primarily based on students’ academic performance. Generally, key high schools are characterized by superior faculty, educational resources, and rigorous pedagogical oversight.

### 2.2 Research tools

#### 2.2.1 Growth mindset scale

Six items from Dweck’s (2006) Mindset Scale were included in the questionnaire to gage a growth mindset. To achieve good reliability and validity, three items were scored negatively (e.g., “Intelligence is difficult to change”), and three items were scored positively (e.g., “No matter who you are, you can greatly change your intelligence”). A 6-point scoring system was employed, where “*completely disagree*” was represented by 1 and “*completely agree*” by 6. In data analysis, three reverse-scored items were reversed, and the average of all six items was calculated to represent the growth mindset score, with higher scores indicating a stronger growth mindset. The coefficient Cronbach’s *α* of this scale in this study is 0.85.

#### 2.2.2 The learning subjective well-being scale for high school students

The Learning Subjective Well-Being Scale for high school students, developed by Ma and Liu (2005), is used to measure learning subjective well-being in high school students. The scale consists of 14 items rated on a 5-point Likert scale ranging from 1 (“*completely agree*”) to 5 (“*completely disagree”*), with higher scores indicating higher levels of learning subjective well-being. The coefficient Cronbach’s *α* of the scale in the study is 0.82.

#### 2.2.3 Achievement motivation scale

The Achievement Motivation Scale, revised by Ye and Hagtvet (1992), was used to measure students’ levels of achievement motivation. The scale consists of 30 items, which can be divided into two dimensions: the pursuit of success (*α* = 0.94, e.g., “I enjoy working persistently to solve problems that I am not confident in”) and avoiding failure (*α* = 0.95, e.g., “I am worried about failure when completing tasks that I consider difficult”). There are 15 items in each dimension, each worth five points. Scores range from 1 (“*completely disagree*”) to 5 (“*completely agree”*), with higher scores indicating stronger motivation. The total score of achievement motivation is the score of the pursuit of success minus the score of the avoidance of failure. The coefficient Cronbach’s *α* of this scale in this study is 0.96.

#### 2.2.4 Grit scale

The Grit Scale, developed by Duckworth et al. (2007), was used to measure students’ grit levels. Translated and revised, the scale comprises 12 items. It employs a 6-point scoring system, ranging from 1 (“*Not at all like me*”) to 6 (“*Very much like me*”). The scale is divided into two dimensions: Perseverance of Effort and Consistency of Interests. The Perseverance of Effort dimension, which includes positively scored items (items 1, 4, 6, 9, 10, and 12, e.g., “I am a hard worker”), has an α of 0.84. The Consistency of Interests dimension, which includes reverse-scored items (items 2, 3, 5, 7, 8, and 11, e.g., “My interests change from year to year”), has an α of 0.80. After reverse scoring the relevant items, the average of all 12 items represents the grit score, with higher scores indicating higher levels of grit. In this study, Cronbach’s *α* coefficient for the scale was 0.78.

## 3 Results

### 3.1 Common method bias test

All data in this study were obtained through questionnaire measurement, so common method bias might exist. The Harman single-factor test was used to assess common method bias (Zhou and Long, 2004). The results showed that there were 10 factors with eigenvalues greater than one, and the explanatory variance of the first principal factor was 24.70%, significantly less than the 40% critical value that was specified. Therefore, the data used in this study do not exhibit any discernible common method bias.

### 3.2 Descriptive statistics and correlation analysis

The descriptive statistical results and correlation of the four variables, namely, growth mindset, learning subjective well-being, grit, and achievement motivation, are shown in Table 2.

**Table 2:** Mean (M), standard deviation (SD), and correlation coefficient of each variable.

	*M*	SD	1	2	3	4	5	6	7	8	9
1.Gender	1.65	0.48	1								
2.Grade	1.95	0.84	0.05	1							
3.Family background	1.35	0.48	−0.05	−0.03	1						
4.Score performance	2.31	0.99	−0.11^**^	−0.20^***^	−0.03	1					
5.School type	1.37	0.48	−0.00	0.09^*^	−0.11^**^	0.05	1				
6.Growth mindset	3.52	1.19	−0.09^*^	−0.06	−0.12^**^	0.03	0.01	1			
7.Achievement motivation	−0.03	0.86	−0.12^**^	0.01	−0.06	−0.11^**^	−0.06	0.27^***^	1		
8.Grit	3.67	0.73	−0.15^***^	0.03	−0.02	−0.13^***^	−0.03	0.30^***^	0.53^***^	1	
9.Learning subjective well-being	3.39	0.63	−0.04	0.01	−0.06	−0.33^***^	−0.06	0.26^***^	0.43^***^	0.51^***^	1

^*^p < 0.05; ^**^p < 0.01; and ^***^p < 0.001, the same hereinafter.For gender, 1 = male and 2 = female. For family background, 1 = countryside and 2 = city. For score performance, 1 = excellent, 2 = good, 3 = average, and 4 = poor. For school type, 1 = key high school and 2 = non-key high school.

There was a significant pairwise positive correlation between a growth mindset, achievement motivation, grit, and learning subjective well-being. Specifically, there was a significant positive correlation (*p* < 0.001) between a growth mindset and achievement motivation, grit, and learning subjective well-being. Learning subjective well-being was correlated with grit (*p* < 0.001). There was also a significant positive correlation (*p* < 0.001) between achievement motivation and learning subjective well-being. Simultaneously, achievement motivation and grit exhibited a strong positive correlation (*p* < 0.001).

### 3.3 Serial mediation model testing

To test the mediating effects of achievement motivation and grit between a growth mindset and learning subjective well-being, we used Model 6 from the SPSS PROCESS macro developed by Hayes (2012). The significance of the mediating effect was tested using a bias-corrected non-parametric bootstrap with 5,000 resamples. Gender, grade, family background, and score performance were included as control variables in the analysis (Wang et al., 2021; Zhang and Lin, 2022).

The results are displayed in Table 3. Regression analysis showed that achievement motivation (*β* = 0.19, *p* < 0.001) and grit (*β* = 0.10, *p* < 0.001) were significantly positively predicted by a growth mindset. Achievement motivation significantly positively predicted grit (*β* = 0.39, *p* < 0.001). After all variables were entered the regression model, a growth mindset still had a significant direct effect on learning subjective well-being (*β* = 0.06, *p* < 0.001). Meanwhile, achievement motivation (*β* = 0.13, *p* < 0.001) and grit (*β* = 0.30, *p* < 0.001) had significant predictive relationships on learning subjective well-being.

**Table 3:** Regression analysis results of serial mediation model.

Result variables	Predictive variables	*R*	*R^2^*	*F*	*β*	*t*	LICI	UICI
Achievement motivation	Growth mindset	0.32	0.10	15.53^***^	0.19	7.11^***^	0.14	0.24
Grit	Growth mindset	0.57	0.32	55.70^***^	0.10	5.22^***^	0.07	0.14
	Achievement motivation				0.39	14.24^***^	0.34	0.45
Learning subjective well-being	Growth mindset	0.61	0.37	59.71^***^	0.06	3.41^***^	0.03	0.09
	Achievement motivation				0.13	5.08^***^	0.08	0.18
	Grit				0.30	9.59^***^	0.24	0.36

The mediating effect was tested, and the results are shown in Table 4. The findings of the mediating effect test indicated that a growth mindset had a significant direct effect on the learning subjective well-being of high school students. Achievement motivation and grit partially mediated the relationship between a growth mindset and learning subjective well-being of high school students. In particular, there were three paths that produced the mediating effect: (1) an indirect path: growth mindset → achievement motivation → learning subjective well-being; (2) an indirect path: growth mindset → grit → learning subjective well-being; (3) an indirect path: growth mindset → achievement motivation → grit → learning subjective well-being. Table 4 shows that the indirect effects of all three paths are significant because none of the paths’ 95% confidence intervals include 0.

**Table 4:** Bootstrap analyses of mediation effect tests (*n* = 708).

	Effect value	95% confidence interval	
Path		Upper limit	Lower limit
Growth mindset → achievement motivation →learning subjective well-being	0.03	0.01	0.04
Growth mindset → grit → learning subjective well-being	0.03	0.02	0.05
Growth mindset → achievement motivation → grit → learning subjective well-being	0.02	0.01	0.03
Direct effect	0.06	0.03	0.09
Indirect effect	0.08	0.06	0.10
Total effect	0.14	0.10	0.17

**Figure 2:**
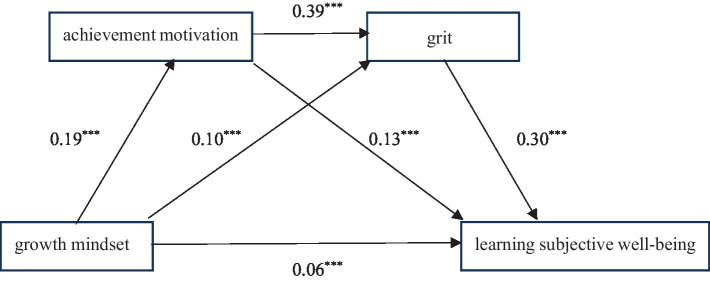
Mediation effect model.

According to the mediation effect model (see Figure 2), the direct path effect from a growth mindset to learning subjective well-being was significant (*β* = 0.06, *p* < 0.001), indicating that the two variables, achievement motivation and grit, partially mediated the relationship between a growth mindset and learning subjective well-being among high school students. Thus, a growth mindset not only predicted learning subjective well-being through grit and achievement motivation these two different paths but also through a serial mediation path from achievement motivation to grit. This proved that the model was a serial mediation model.

## 4 Discussion

By building a serial mediation model, this study thoroughly examined the combined effects of a growth mindset, achievement motivation, and grit on the learning subjective well-being of high school students. The primary focus was on how achievement motivation and grit mediated the relationship between a growth mindset and learning subjective well-being in high school students, which helped to strengthen our comprehension of the relationship between a growth mindset and learning subjective well-being

### 4.1 Associations of a growth mindset and learning subjective well-being

Consistent with our first hypothesis, the results of this study indicated that a growth mindset positively predicted high school students’ learning subjective well-being. This finding aligns with previous studies (Ortiz Alvarado et al., 2019; Liu et al., 2021), suggesting that a growth mindset has a beneficial impact on subjective well-being. Specifically, our study further focuses on subjective well-being in the specific domain of learning, rather than the more general subjective well-being that most studies explore. Furthermore, this result enriches the mindset theory perspective, which posits that people who have a growth mindset believe that abilities are malleable and can be developed through hard work paid in learning (Zeng et al., 2019; Liu, 2020). Individuals who are more growth-minded tend to have greater confidence in their abilities for development. They will actively learn, seek strategies, improve their abilities, and will not back down from challenges or give up easily (Blackwell et al., 2007). Meanwhile, according to Dweck (2006), students who have a strong growth mindset tend to set manageable goals. Their learning subjective well-being is higher because they focus on developing abilities and mastering knowledge and skills. They dare to try, enjoy the process of learning and hard work, and accumulate more positive experiences. Therefore, we should focus on fostering a growth mindset among high school students to enhance their learning subjective well-being. It will also promote their all-round development and lay a strong foundation for future life planning and development. By deeply exploring emotional experiences, motivation, and satisfaction in the learning process, we aim to uncover key factors influencing students’ subjective well-being. This not only aids in enhancing educational outcomes but also provides valuable insights for policymakers, promoting more human-centered educational reforms emphasized within the context of Chinese education.

### 4.2 The mediating role of achievement motivation and grit

The findings of the study demonstrated that achievement motivation and grit significantly mediated the relationship between a growth mindset and learning subjective well-being. Achievement motivation served as a mediating variable between a growth mindset and learning subjective well-being of high school students, and hypothesis 2 is validated. This aligns with the conclusion of previous research (Gao and Bai, 2021; Zhao et al., 2022). For instance, in the university student population, a growth mindset predicted achievement motivation (Zhao et al., 2022). Achievement motivation was significantly positively correlated with subjective well-being (Gao and Bai, 2021), and this result is in line with the views of the Intellectual Achievement Motivation Model and the Goal Theory of Happiness (Brunstein and Schmitt, 2004; Dweck, 2006). Individuals with different mindsets set different achievement goals, and different goal orientations lead to different motivations. Those who possess a strong growth mindset set achievable goals, signifying a stronger motivation to pursue success. Learning subjective well-being is produced when personal needs are met or goals are achieved. People with stronger growth mindsets tend to have more confidence in their potential, believing that with practice and education, they can achieve greater development, overcome difficulties, and complete tasks, thus having a stronger motivation for achievement. The generation of achievement motivation drives individuals to work harder, and when they achieve some results, their self-actualization and achievement needs are met (Song et al., 2015; Gao and Bai, 2021). So, the entire process generates more positive emotions and happiness, eventually raising higher learning subjective well-being. Previous studies have separately described relationships between two variables, such as a growth mindset and achievement motivation (Zhao et al., 2022), or achievement motivation and subjective well-being (Gao and Bai, 2021). In contrast, our study integrates these three variables into a mediation chain and employs appropriate theories to explain the relationships among them. This represents a novel aspect of our research. This study is one of the few studies to apply such theories (i.e., the Intellectual Achievement Motivation Model and the Goal Theory of Happiness) in the context of a growth mindset and learning subjective well-being by demonstrating that adolescents who hold a belief in the malleability of abilities in real life tend to set achievement goals and strive relentlessly to achieve them, which may lead to higher levels of well-being in the learning process.

The research results also indicated that grit was another mediating variable between a growth mindset and learning subjective well-being of high school students, confirming hypothesis 3. This result is consistent with previous research (Liu et al., 2021). For instance, a growth mindset indirectly influenced subjective well-being through grit, while subjective well-being was indirectly measured via life satisfaction and positive and negative emotions (Liu et al., 2021). In contrast, our study employed the High School Students’ Learning Subjective Well-Being Scale for a more direct measurement. Moreover, the finding aligns with Dweck’s (2006) perspective on mindset. Individuals with a growth mindset believe in the significance of hard work, constantly strive toward their goals, and possess higher grit. In addition, this result corroborates the character strengths theory (Wagner and Ruch, 2015; Jiang et al., 2018), as grit, being a positive personality trait, manifests its advantageous effects through individual cognition, emotions, and behavior. Our study extends the application scope of character strengths theory by linking grit with learning well-being, whereas previous research primarily focused on the relationship between grit and academic performance based on this theory (Wagner and Ruch, 2015; Jiang et al., 2018). This finding facilitates sustained academic development in high school students, fostering a positive attitude toward learning. To sum up, high school students with a growth mindset tend to persevere and not easily give up when facing challenging tasks, enjoying the process of hard work (Dweck, 2006), and thus experiencing higher learning subjective well-being.

Finally, when studying the mechanism of the relationship between a growth mindset and learning subjective well-being, a serial mediation path from achievement motivation to grit was also discovered, validating Hypothesis 4. Grit can be positively predicted by achievement motivation, which is consistent with previous research findings (Li et al., 2021; Zhao et al., 2022). This result is also consistent with the achievement motivation theory (McClelland, 1961). Individuals with a strong need for achievement, driven by achievement motivation, demonstrate sustained effort and perseverance when completing tasks. They continuously overcome difficulties, aspire to complete tasks more perfectly, and strive for success (Li and Lv, 2022; Zhao et al., 2022). Achievement motivation generally manifests as an individual’s effort and persistence in the task. The stronger the achievement motivation, the higher the grit displayed when facing tasks and difficulties. Overall, high school students with stronger growth mindset believe that it is possible to develop abilities, have stronger achievement motivation, have more grit in facing challenges in their academic and personal lives, experience more joyful emotions, and have higher learning subjective well-being. Our study aims to elucidate the underlying mechanisms between a growth mindset and learning subjective well-being by validating the mediating roles of achievement motivation and grit. While some studies have emphasized similar themes (Hassan et al., 2023; Valdez, 2024), our research focuses more specifically on subjective well-being in the learning context within the Chinese cultural background. Creatively exploring the separate and sequential mediating roles of achievement motivation and grit, we reveal the intricate mechanisms through which a growth mindset influences learning subjective well-being. This provides further theoretical and empirical support for educational practices within the Chinese cultural context

### 4.3 Contributions and implications

This study explores the unique impact of a growth mindset on the subjective well-being of high school students and elucidates its internal mechanisms, thereby filling a research gap. Theoretically, we enrich the theoretical framework concerning the relationship between psychological factors and subjective well-being in learning by demonstrating how a growth mindset indirectly influences learning well-being through achievement motivation and grit. In practical terms, our findings provide valuable guidance for educational practices.

First, teachers should pay attention to changing students’ fixed mindsets from a cognitive perspective. They can achieve this by conveying the concept of developmental abilities to them, promoting the internalization of ability plasticity through strategies such as writing guidance letters, and helping students establish a growth mindset (Zhang et al., 2021).

Second, interventions aimed at enhancing learning subjective well-being should guide high school students to establish appropriate achievement motivation and improve motivation structure. For example, teachers can guide students to establish mastery goals, allowing them to experience pride, satisfaction, and other positive emotions in learning by achieving small, incremental objectives (Gao and Bai, 2021).

Third, developing the grit of high school students helps to enhance their learning subjective well-being. Teachers can teach students to understand failure and setbacks correctly, praise their efforts rather than just focusing on the results, and encourage them to be resilient and brave (Su et al., 2021; Zhang et al., 2021).

Finally, our research findings indicate that serial mediators link a growth mindset with learning subjective well-being. Therefore, emphasizing the development of achievement motivation and grit as mediators in education and teaching may be more effective than employing one mediator. Educational and teaching intervention plans should promote the development of achievement motivation and grit in the process of developing a growth mindset among high school students.

### 4.4 Limitations and future recommendations

This study has the following limitations. (1) All research data were collected through questionnaires, and a cross-sectional design was used. Since it is challenging to ascertain causal relationships with a cross-sectional design, longitudinal observations and design intervention experiments can be used in future research to thoroughly explore causal relationships. (2) This study used the self-report method to measure the growth mindset, achievement motivation, grit, and learning subjective well-being of participants. Participants’ responses may be less authentic or objective due to social approval influence. More multi-source, objective measurement techniques may be used in subsequent studies. (3) The study employed the convenience sampling method, which may have introduced sampling error but still partially captured the features of the target population. In the future, studies could explore employing a random sampling technique with a larger sample size to improve the external validity of the study and better represent the target population.

## 5 Conclusion

This study offers valuable insights into the relationship between a growth mindset, achievement motivation, grit, and learning subjective well-being of high school students. The study concludes that (1) a growth mindset positively predicted learning subjective well-being among high school students. (2) Achievement motivation played a mediating role between a growth mindset and learning subjective well-being among high school students. (3) Grit played a mediating role between a growth mindset and learning subjective well-being among high school students. (4) Achievement motivation and grit played a serial mediation role between a growth mindset and learning subjective well-being among high school students. By gaining a deeper understanding of the influence mechanisms underlying the learning subjective well-being of high school students, educators can promote their growth mindset, achievement motivation, and grit by actively guiding and setting attainable goals, thereby enhancing their learning subjective well-being.

## Data availability statement

The raw data supporting the conclusions of this article will be made available by the authors, without undue reservation.

## Ethics statement

The studies involving humans were approved by the ethics committee of Zhejiang Normal University. The studies were conducted in accordance with the local legislation and institutional requirements. Written informed consent for participation in this study was provided by the participants’ legal guardians/next of kin.

## Author contributions

WZ: Writing – review & editing, Methodology, Data curation, Writing – original draft, Investigation, Conceptualization. XS: Conceptualization, Writing – review & editing, Writing – original draft, Investigation, Data curation. MJ: Writing – review & editing, Methodology, Conceptualization. YL: Writing – review & editing, Methodology, Conceptualization. CL: Writing – review & editing, Data curation. YJ: Writing – review & editing, Investigation. JC: Writing – review & editing, Investigation. MO: Writing – review & editing. XL: Writing – review & editing. YT: Writing – review & editing.
